# Ocular Manifestations and Neuropathy in Type 2 Diabetes Patients With Charcot Arthropathy

**DOI:** 10.3389/fendo.2021.585823

**Published:** 2021-04-21

**Authors:** Marilia Trindade, Jessica Castro de Vasconcelos, Gabriel Ayub, Alex Treiger Grupenmacher, Delma Regina Gomes Huarachi, Marina Viturino, Maria Lucia Correa-Giannella, Yeelen Ballesteros Atala, Denise Engelbrecht Zantut-Wittmann, Maria Candida Parisi, Monica Alves

**Affiliations:** ^1^ Department of Ophthalmology, School of Medical Sciences, University of Campinas (UNICAMP), Campinas, São Paulo, Brazil; ^2^ Endocrinology Division, Department of Internal Medicine (Endocrinology), School of Medical Sciences, University of Campinas (UNICAMP), Campinas, São Paulo, Brazil; ^3^ Programa de Pos-Graduação em Medicina, Universidade Nove de Julho (UNINOVE), São Paulo, Brazil; ^4^ Laboratório de Carboidratos e Radioimunoensaio (LIM-18), Hospital das Clínicas HCFMUSP, Faculdade de Medicina da Universidade de São Paulo, São Paulo, Brazil

**Keywords:** ocular surface, diabetes, Charcot arthropathy, neuropathy, dry eye, cardiovascular autonomic neuropathy

## Abstract

**Objective:**

Diabetes can affect the eye in many ways beyond retinopathy. This study sought to evaluate ocular disease and determine any associations with peripheral neuropathy (PN) or cardiac autonomic neuropathy (CAN) in type 2 diabetes (T2D) and Charcot arthropathy (CA) patients.

**Design:**

A total of 60 participants were included, 16 of whom were individuals with T2D/CA, 21 of whom were individuals with T2D who did not have CA, and 23 of whom were healthy controls. Ocular surface evaluations were performed, and cases of dry eye disease (DED) were determined using the Ocular Surface Disease Index (OSDI) questionnaire, ocular surface staining, Schirmer test, and Oculus Keratograph 5M exams. All variables were used to classify DED and ocular surface disorders such as aqueous deficiency, lipid deficiency, inflammation, and ocular surface damage. Pupillary and retinal nerve fiber measurements were added to the protocol in order to broaden the scope of the neurosensory ocular evaluation. PN and CAN were ascertained by clinical examinations involving the Neuropathy Disability Score (for PN) and Ewing’s battery (for CAN).

**Results:**

Most ocular variables evaluated herein differed significantly between T2D patients and controls. When the controls were respectively compared to patients with T2D and to patients with both T2D and CA, they differed substantially in terms of visual acuity (0.92 ± 0.11, 0.73 ± 0.27, and 0.47 ± 0.26, p=0.001), retinal nerve fiber layer thickness (96.83 ± 6.91, 89.25 ± 10.44, and 80.37 ± 11.67 µm, p=0.03), pupillometry results (4.10 ± 0.61, 3.48 ± 0.88, and 2.75 ± 0.81 mm, p=0.0001), and dry eye symptoms (9.19 ± 11.71, 19.83 ± 19.08, and 24.82 ± 24.40, p=0.03). DED and ocular surface damage also differed between individuals with and without CA, and were associated with PN and CAN.

**Conclusion:**

CA was found to be significantly associated with the severity of ocular findings. DED in cases of CA was also associated with PN and CAN. These findings suggest that intrinsic and complex neurosensory impairment in the eyes, peripheral sensory nerves, and the autonomic nervous system are somehow connected. Thus, a thorough ocular evaluation may be useful to highlight neurological complications and the impact of diabetes on ocular and systemic functions and structures.

## Introduction

Chronic complications of type 2 diabetes (T2D) are progressive, simultaneous, and regulated by both genetic predisposition and environmental factors (1). Understanding all mechanisms involved in their pathogenesis and implications remains a challenge. Charcot arthropathy (CA) is a serious debilitating complication of diabetes mellitus that can occur in 0.4%–13% of cases, increasing morbidity and mortality among diabetic patients (2). Several authors consider it the most devastating complication of diabetes (3, 4). The role of motor, sensory, and peripheral neuropathy in CA has been largely studied, but there are still factors to be explored. Another complication associated with CA is diabetic autonomic neuropathy, in which neurosensory damage initially affects parasympathetic nerve fibers, resulting in autonomic imbalance, increased sympathetic activity, and decreased vagal function. One form of diabetic autonomic neuropathy is cardiovascular autonomic neuropathy. It is considered extremely serious as it increases the risk of stroke, perioperative morbidity, and silent myocardial ischemia (5, 6).

CA diagnosis is based on patient’s history and clinical signs and is confirmed radiologically. CA affects the bones, joints, and soft tissues of the foot and ankle and may be caused by sensory-motor neuropathy, autonomic neuropathy, trauma, or metabolic bone abnormalities related to diabetes (7–9). Pathophysiological mechanisms of CA are complex and remain unclear. They include peripheral and autonomic neuropathies with high blood flow to the foot that lead to increased bone resorption (10). The condition may also involve peripheral somatic polyneuropathy with loss of protective sensation and high risk of unrecognized acute or chronic minor trauma (11, 12). In both cases, there is an excessive local inflammatory response to foot injury that results in local osteoporosis (13). In the acute stage, CA is characterized by a hot, swollen foot; in the chronic stage, it is represented by local inflammation and progressive bone disruption and destruction associated with sensory neuropathy with loss of protective sensation. It ultimately causes deformities and increases the risk of foot ulceration (14, 15).

Diabetic retinopathy is a prevalent chronic complication of diabetes but is not the only ocular manifestation of the disease. The ocular surface may also be affected, such as in dry eye disease (DED) (16–18). The prevalence of DED among T2D patients has been reported to be as high as 54.3% (16). Variations in DED symptoms can make a diagnosis challenging. Some patients experience a highly symptomatic condition that impacts their quality of life and vision, while other patients with neurosensory abnormalities, even profound ocular surface damage, may be asymptomatic as they progress to vision-threatening complications. DED can induce visual disturbances and loss of ocular surface homeostasis, which, in turn, may generate corneal epithelial defects, erosions, or ulcers (16).

Peripheral neuropathy is the most common diabetic neuropathy. It may manifest with sensory and motor deficits (19–22). The ocular surface is densely innervated, and this innervation is essential to maintain tear secretion, epithelial renovation, and blinking and to guarantee homeostasis. Thus, any neuropathic disruptions may profoundly impact tear production, ocular surface integrity, comfort, and quality of vision (23–26). Data suggest that patients with peripheral neuropathy also have reduced corneal sensitivity (16), and a rarefication of corneal sub-basal nerve plexus including patients with early stage of CA (25). Indeed, the International Dry Eye Workshop II (DEWS II) report recently included neurosensory abnormalities in the definition of DED in the form of decreased reflex-induced lacrimal secretion, a lower blink rate, and increased evaporative tear loss (22, 27, 28). Although peripheral neuropathy and DED are common among diabetes patients, the relationship between them is not completely understood.

Chronic complications can happen concomitantly and vary in accordance with patients’ genetic profiles. In this context, this study sought to determine the ways in which ocular findings are associated with autonomic and peripheral neuropathies in T2D patients with and without CA relative to healthy controls.

## Materials and Methods

### Study Design

This was a cross-sectional, observational, and non-interventional study carried out between January 2019 and September 2019. The three groups consisted of one group with subjects with T2D and CA (the T2D+CA Group), a second group of patients with T2D but who did not have CA (the T2D Group), and a control group of healthy individuals. In addition to the clinical data of interest in this study, laboratory and demographic data were collected during clinical and ophthalmological consultations, which were performed at a tertiary center in the city of Campinas, São Paulo, Brazil. This study was submitted to and received approval from the local ethics research committee.

### Subjects

T2D was diagnosed based on plasma glucose criteria used at diagnosis time (29, 30). CA was diagnosed based on radiological criteria (joint congruence, bone destruction, talar-first metatarsal angle, flatfoot) and clinical diagnostic criteria such as vascular conditions (hyperemia, edema, comparative temperature), neuropathy (pain, proprioception, dehydration), osteoarticular abnormalities (equinus, clawed toes, instability), and cutaneous abnormalities (ulcer, hyperkeratosis, infection) (31). The three groups were similar in terms of age and sex (all subjects were 18 years of age or older), and all subjects voluntarily agreed to participate. Patients experiencing acute complications of diabetes at the time of their exam and patients with glaucoma, high axial myopia, ocular trauma, contact lens wearer, chronic topical steroids users, tilted disc, type 1 diabetes or gestational diabetes, stage 3 chronic kidney disease or higher, end-stage kidney disease, arrhythmia, or severe illnesses such as heart failure, presence of rheumatological and immunological diseases, liver cirrhosis, alcoholism, severe infection, or malignancy were excluded.

### Ocular Assessment

DED symptoms were evaluated using the Ocular Surface Disease Index (OSDI) questionnaire. It consists of 12 items that assess symptoms, functional limitations, and environmental factors, and patients score each item from 0 (symptoms none of the time) to 4 (symptoms all the time). The total score ranges from 0 to 100. A score between 0 and 12 is considered normal, while a score of 13 to 22 reflects mild DED, a score of 23 to 32 represents moderate DED, and a score of 33 or above indicates severe DED (32).

**Figure 1:**
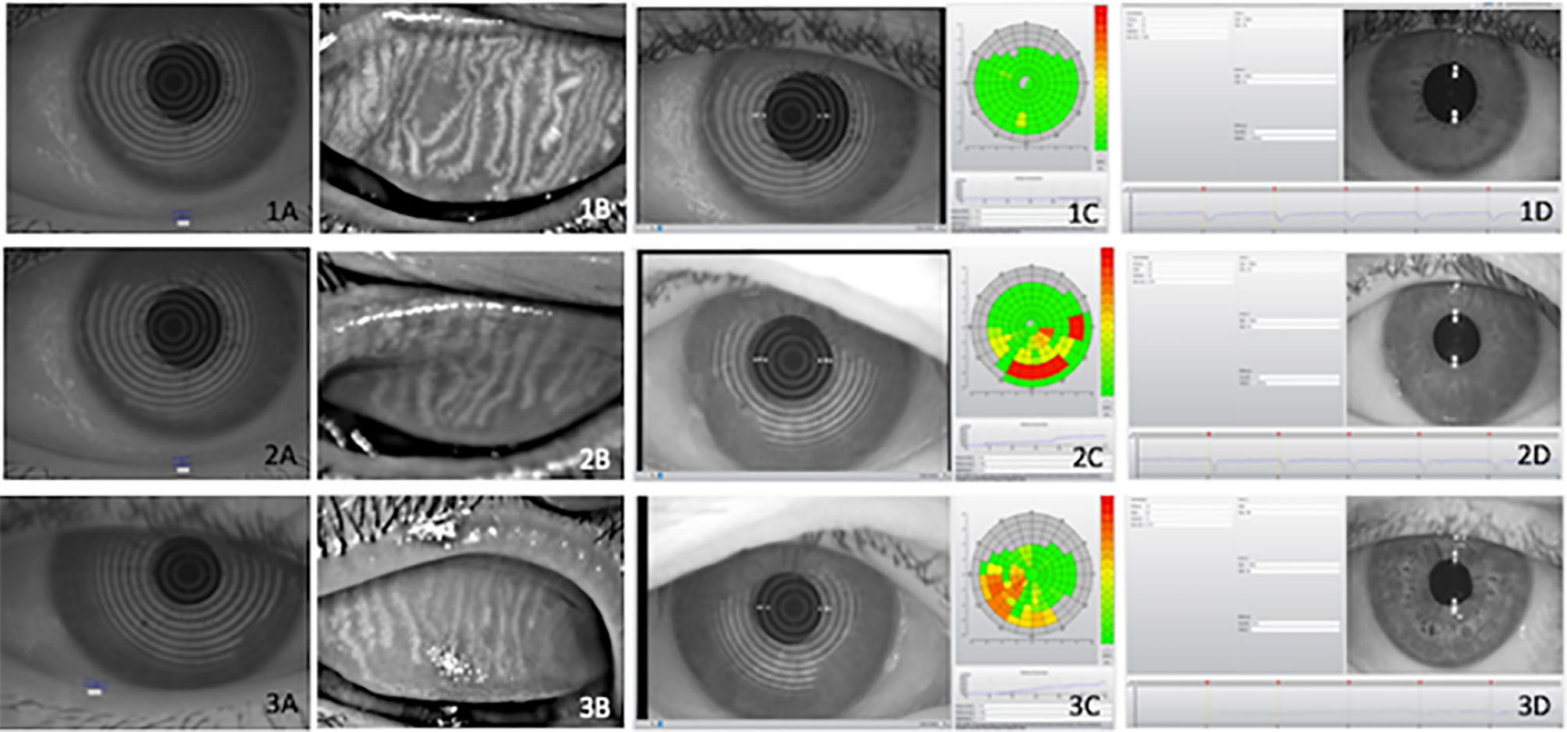
Illustrative examples of objective ocular parameters in control (1), type 2 diabetes (T2D) patients (2) and diabetic with Charcot arthropathy (3) Tear meniscus height **(A)**, meibography **(B)**, Noninvasive Break Up Time **(C)** and pupillometry **(D)**. Ocular parameters in (1) heathy individuals; (2) type 2 diabetes (T2D) and (3) patients with Charcot arthropathy. Tear meniscus height **(A)**; meibography **(B)**; Non-invasive Break Up Time **(C)** and pupillometry **(D)**.

The ocular surface evaluation consisted of meibography, pupillometry, meniscometry, non-invasive tear film break-up time measurement, and conjunctival hyperemia quantification, all of which were performed using the Oculus Keratograph 5M (OCULUS Optikgerate GmbH, Wetzlar, Germany) followed by ocular surface staining with fluorescein and lissamine, and Schirmer test without anesthesia (**Figure 1**). All procedures were performed by the same examiner using the techniques described below:

Tear film stability was assessed in two different ways. First, non-invasive tear film break-up time (NITBUT) was determined using the Keratograph 5M and through the evaluation of Placido concentric rings during continuous eye-opening intervals. Next, fluorescein tear film break-up time (TBUT) was measured by administering 5 μl of a 2% sodium fluorescein solution (Allergan, Guarulhos, São Paulo, Brazil) and calculating the average of three consecutive break-up times, determined manually using a stopwatch. Tear meniscus height was measured in millimeters on images taken by the Keratograph 5M.

To assess meibomian gland function, non-contact infrared meibography was performed on the lower and upper lids using the Keratograph 5M. The meiboscore, the classification scale adapted from Arita et al. (33), uses the following scale for each eyelid: 0 (no loss of meibomian glands); 1 (loss of the meibomian gland involving less than one third of the total meibomian gland area); 2 (a loss between one third and two thirds of the total area of ​​the meibomian gland); and 3 (a loss of more than two thirds of the total meibomian gland area).

Conjunctival hyperemia was graded as follows: 0, absent; 1, mild; 2, moderate; and 3, severe. Corneal staining was recorded and graded on the Oxford Scheme. Dry eye severity scores from 1 to 4 were based on the DEWS classification.

### Classification of Ocular Surface and Tear Film Dysfunction

To better understand the correlations and possible mechanisms involved in ocular surface dysfunction and diabetes, all exam results were compiled and combined. Ocular test results were used to summarize the impact of diabetes on ocular surface homeostasis, a change in which was determined by cases of aqueous deficiency, lipid deficiency, ocular surface damage, and inflammation. Tear deficiency was defined as the sum of Schirmer test and tear meniscus height results. Lipid deficiency/evaporative dry eye was based on meiboscore and TBUTs. Inflammation was determined using the conjunctival hyperemia and OSDI results, and ocular surface damage was defined by lissamine and fluorescein staining scores. In other words, surface damage represents the sum of fluorescein and lissamine staining, lipid deficiency is the sum of NITBUT and meibography results, and tear deficiency is the sum of Schirmer test and tear meniscus height.

### Ocular Neurosensorial Parameters

Pupillometry and retinal nerve fibers were evaluated as possible parameters of ocular neurosensorial dysfunction. Pupillometry assessed the pupillary reflex at two different glare stimulus powers and without glare using automatic measurement (Keratograph 5M). Graphic results show pupil changes over a period of time, according mean pupil diameter (measured in millimeter), followed by the standard deviation (34). Central thickness of the retinal nerve fiber layer (RNFL) was measured using optical coherence tomography (Heidelberg Spectralis OCT, glaucoma module, peripapillary retinal and RNFL circular scan, 100 frames and 768 A-scans).

#### Neuropathy Assessment

Peripheral diabetic neuropathy was diagnosed by the Neuropathy symptom score (NSS) and by the Neuropathy disability score (NDS). NSS explores pain or discomfort in the legs (burning, numbness or tingling, fatigue, cramping), the presence of symptoms in the feet, calves or elsewhere, nocturnal exacerbation of symptoms, both day and night or daytime alone, if the symptoms had ever woken the patient from sleep. The patients were asked if any maneuver could reduce the symptoms: walking, standing, sitting or lying down. On the other hand, Neuropathy NDS was derived from examination of the knee and ankle reflex, feet sensation with the Semmes-Weinstein 5.07 [10 g] filaments and vibration (35).

Cardiovascular autonomic neuropathy was diagnosed using the cardiovascular autonomic reflex tests (CARTs), also known as Ewing’s battery. These determine heart rate in response to deep breathing (expiration-to-inspiration [E:I] ratio), to the Valsalva maneuver, and to lying-to-standing tests (orthostatic test, 30:15 ratio). The CARTs consider changes in blood pressure (BP) after standing. While heart rate changes during the former three tests mainly reflect parasympathetic function, BP in the Valsalva maneuver and orthostatic hypotension reflect sympathetic function (36). Heart rate variability (HRV) in time and frequency domain indices have been described as another tool to evaluate cardiovascular autonomic neuropathy (37). CARTs and HRV are frequently used for cardiovascular autonomic neuropathy diagnosis because they exhibit good reproducibility, are easy to execute, and, when combined, provide high specificity (38, 39).

#### Statistical Analysis

Exploratory data analysis was performed through summary measures (mean, standard deviation, minimum, median, maximum, frequency, and percentage). Comparisons between groups were performed using the Wilcoxon test. The correlation between numerical variables was assessed using Spearman’s rank correlation coefficient. The level of significance was 5%. The analyses were performed using the Statistical Analysis System (SAS) for Windows, version 9.4 (SAS Institute Inc., Cary, NC, USA).

## Results

This study included a total of 60 individuals between 27 and 77 years of age (58.98 ± 9.63) who were divided into the T2D+CA Group (n=16), the T2D Group (n=21), and the control group (n=23). The two groups that included T2D patients were similar for disease duration and there were no statistically significant differences as far as sex is concerned among the groups. All CA patients had peripheral neuropathy and cardiovascular autonomic neuropathy. Most T2D patients who did not have CA were diagnosed with either peripheral neuropathy (86%) or cardiovascular autonomic neuropathy (76%). No individuals from the control group had peripheral neuropathy or cardiovascular autonomic neuropathy. **Table 1** presents the distribution of the groups and the association between autonomic and peripheral neuropathies.

**Table 1:** Comparative analysis among the groups of type 2 diabetes (T2D) patients with Charcot arthropathy (CA), type 2 diabetes patients without CA, and healthy individuals according to autonomic and peripheral neuropathies.

Variables		T2D Group (n=21)	T2D + CA Group (n=16)	Control Group (n=23)	P Value^†^
Sex	Male	10	11	9 (39.13%)	0.184
		(47.62%)	(68.75%)		
	Female	11 (52.38%)	5 (31.25%)	14 (60.87%)	
Age (in years)		60.62 ± 7.91	57.12 ± 11.76	58.78 ± 9.61	0.664
Peripheral Neuropathy	No	3 (14.29%)	0 (0.00)	23 (100%)	<0.0001^a, b, c^
	Yes	18 (85.71%)	16 (100.00)	0 (0.00)	
CAN	Absent	5 (23.81%)	0 (0.00)	23 (100%)	<0.0001^a, b, c^
	Incipient	3 (14.29%)	5 (31.25%)	0 (0.00)	
	Established	13(61.90%)	11 (68.75%)	0 (0.00)	

CAN, Cardiovascular autonomic neuropathy.

Data expressed as mean ± standard deviation or frequency.

^†^Chi-square test.

p value for the comparison of (^a^) T2D with controls (^b^) T2D+CA with controls and (^c^) T2D with and without CA.

The study subjects’ clinical, metabolic, and demographic profiles are presented in **Table 2**. There were no significant differences in glycated hemoglobin (HbA1c) between the subjects. Kidney function was significantly lower among T2D patients with CA relative to T2D patients who did not have CA.

**Table 2:** Comparative analysis of clinical, demographic, and metabolic characteristics of type 2 diabetes (T2D) patients with and without Charcot arthropathy (CA).

Variables	T2D Group (n=21)	T2D + CA Group(n=16)	P Value
HbA1c (%)	8.68 ± 1.48	8.45 ± 2.18	0.5625^††^
Creatinine (mg/dl)	0.92 ± 0.23	1.28 ± 0.42	0.014^††^
GFR (mL/min/1.73 m ^2^	84.49 ± 20.96	64.99 ± 23.65	0.012^††^
Diabetes duration (in years)	16.19 ± 8.67	19 ± 10.14	0.61^††^
Diagnosis of dyslipidemia	43%	57%	0.02^†^
SAH	62%	47%	0.04^†^
Smoking	9.5%	33.3%	<0.0001^†^
PDR	14.3%	42.8%	<0.001^†^

Data expressed as mean ± standard deviation or frequency.

^†^Chi-square test.

^††^Mann-Whitney test.

GFR, Glomerular filtration rate; SAH, Systemic arterial hypertension;

PDR, Proliferative diabetic retinopathy.

Subjects with T2D had significantly more abnormalities in their ocular surface variables indicative of DED and ocular surface dysfunction relative to the control group. Indeed, ocular neurosensory pathways such as central thickness of the RNFL and pupillary diameter were abnormal in subjects with T2D (T2D + CA Group > T2D Group > Control Group). All results of the ocular parameters were the most abnormal among patients with both T2D and CA. **Table 3** provides the ocular variable information from each group.

**Table 3:** Comparisons in ocular variables between type 2 diabetes (T2D) patients with Charcot arthropathy (CA), T2D patients without CA, and healthy individuals.

Variables	T2D Group (n=21) mean ± SD	T2D + CA Group (n=16) mean ± SD	Control Group(n=23) mean ± SD	P Value^††^
Visual Acuity	0.73 ± 0.27	0.47 ± 0.26	0.92 ± 0.11	0.0001^a,b,c^
RNFL (µm)	89.25 ± 10.44	80.37 ± 11.67	96.83 ± 6.91	0.0315^b^
Pupillometry (mm)	3.48 ± 0.88	2.75 ± 0.81	4.10 ± 0.61	0.0001^a, b, c^
Tear meniscus height (mm)	0.24 ± 0.08	0.28 ± 0.10	0.30 ± 0.08	0.0564
NITBUT (seconds)	9.34 ± 6.82	7.59 ± 4.48	13.39 ± 7.00 (11.09)*	0.0205^a, b^
Hyperemia (grade 0-4)	1.83 ± 0.66	1.72 ± 0.56	1.69 ± 0.52	0.8076
Fluorescein (grade 0-15)	0.81 ± 0.98	3.37 ± 3.50	0.30 ± 0.56	0.0031^a, c^
Lissamine (grade 0-4)	1.50 ± 1.79	1.00 ± 1.15	0.35 ± 0.57	0.0330^a, b^
Schirmer test (mm)	13.76 ± 9.99	13.06 ± 10.04	11.48 ± 4.66	0.9363
Upper lid meiboscore (grade 0-4)	1.35 ± 0.81	1.62 ± 0.72	0.91 ± 0.79	0.0330^b^
OSDI (grade 0-100)	19.83 ± 19.08	24.82 ± 24.40	9.19 ± 11.71	0.0367^a, b^

Data expressed in mean ± standard deviation or median*.

^††^Kruskal-Wallis test.

RNFL, Retinal nerve fiber layer.

OSDI, Ocular surface disease index (OSDI).

NITBUT, Non-invasive tear break-up time.

p value for the comparison of (^a^) T2D with controls (^b^) T2D+CA with controls and (^c^) T2D with and without CA.

A relevant association was found between ocular surface disease, T2D, and CA. Ocular surface damage and lipid deficiency were more frequent in the T2D + CA Group. On a three-point scale between absent and established, the established form of tear deficiency was more frequent among T2D subjects (**Table 4**). Some subjects in the control group presented incipient ocular surface disease, which may rely on aging processes (another relevant risk factor for this condition). In the comparisons between distinct neuropathies in patients with T2D, peripheral neuropathy was found to be associated with surface damage (p = 0.0030), lipid deficiency (p = 0.0030), and tear deficiency (p < 0.0001). Cardiovascular autonomic neuropathy was also found to be associated with surface damage (p = 0.0040), lipid deficiency (p = 0.0020), and tear deficiency (p = 0.0180). All test results were the most abnormal among patients with both T2D and CA (T2D + CA Group > T2D Group > Control Group). **Figure 1** provides illustrative images of objective parameters in healthy individuals, type 2 diabetes (T2D) patients and diabetic with Charcot arthropathy. Tear meniscus height to infer aqueous tear volume, meibography to evaluate lipid layer production glands, Noninvasive Break Up Time to measure tear stability and also pupillometry.

**Table 4:** Comparison of Ocular surface dysfunction between type 2 diabetes (T2D) patients with Charcot arthropathy (CA), T2D patients without CA, and healthy individuals.

	T2D Group(n=21)	T2D + CAGroup(n=16)	ControlGroup(n=23)	P Value^†^
Surface Damage				0.0003 a, b
Absent	1 (4.76)	6 (37.50)	21 (91.30)	
Incipient	8 (38.67)	5 (31.25)	2 (8.70)	
Established	12 (57.14)	5 (31.25)	0 (0.00)	
Lipid Deficiency				<0.0001^a, b^
Absent	2 (9.52)	0 (0.00)	9 (39.13)	
Incipient	7 (33.33)	3 (18.75)	11 (47.83)	
Established	12 (57.14)	13 (81.25)	3 (13.04)	
Tear Deficiency				<0.0001^a, b^
Absent	2 (9.52)	5 (31.25)	14 (60.87)	
Incipient	2 (9.52)	6 (37.50)	9 (39.13)	
Established	12 (57.14)	5 (31.25)	0 (0.00)	

^†^Chi-square test.

p value for the comparison of (^a^) T2D with controls (^b^) T2D+CA with controls and (^c^) T2D with and without CA.

## Discussion

This study evaluated the associations between different types of neuropathy (namely, peripheral neuropathy and cardiovascular autonomic neuropathy) and ocular findings in T2D patients with CA, T2D patients without CA, and healthy individuals. Almost all of the ocular variables differed significantly between the three groups. The T2D + CA Group exhibited more abnormal results than the T2D Group, which, in turn, exhibited more negative results than the control group.

In the T2D + CA Group, all of the patients had peripheral neuropathy and cardiovascular autonomic neuropathy. Meanwhile, their respective frequencies in the T2D Group were 86% and 76%. The rate of cardiovascular autonomic neuropathy in this study was higher than the 20% among European T2D patients reported by Spallone et al. (40). The relatively high rate seen herein may be the result of the current study’s inclusion of a small number of subjects, and of patients who were from a tertiary hospital, had a longer mean T2D duration, and, in some cases, had CA.

RNFL loss occurs in patients with diabetes regardless of diabetic retinopathy, suggesting that the function of neuronal cells in the retina is compromised even before the appearance of microvascular changes (14, 41, 42). The OCT imaging applied herein showed that RNFL thickness decreased gradually in the three groups and was the most impaired in the T2D + CA Group. Several studies have shown differences in RNFL thickness between individuals with diabetes and a healthy age-matched population (38). Additionally, previous reports have shown full retinal and inner retinal thickness to be significantly reduced in individuals with neuropathy, particularly among patients at increased risk of foot ulceration (43). RNFL thinning represents indirect evidence of diabetes-induced neurodegeneration, which may precede the development of diabetic retinopathy.

A typical manifestation of diabetic autonomic neuropathy is pupillary autonomic neuropathy, which affects pupillary function (44). Pupillometry can be used to assess the integrity of afferent visual pathways and to determine the balance between the sympathetic constrictor and parasympathetic dilator systems (45). Wang et al. emphasizes that pupil dilation requires both parasympathetic and sympathetic innervation of the iris (46). The current study revealed a smaller mean pupil diameter in both experimental groups and particularly in the T2D + CA Group. These results indicate both parasympathetic and sympathetic autonomic dysfunction and reinforce the utility of pupillometry in determining the presence or absence of autonomic neuropathy (44).

DED can occur in diabetics as a result of decreased corneal sensitivity associated with the development of diabetic neurotrophic keratopathy (47), while decreased tear film stability results from decreased goblet cell density (48). The combination of lacrimal gland function assessment and Schirmer test has determined lower tear production rates in diabetics than in non-diabetic individuals, except in initial compensatory phases of DED (21). In T2D patients, ocular surface changes (including reduced tear film stability and secretion, reduced sub-basal nerve density, and reduced corneal sensitivity) can occur simultaneously and even prior to clinical evidence of peripheral or autonomic neuropathy. The subjects included herein exhibited broad impacts of ocular surface disease as determined by the assessments of tear stability (NITBUT), epithelial integrity (fluorescein staining), lipid production (meiboscore), and symptom intensity (OSDI), all of which were found to represent gradual impairment according to the severity of the disease and its complications, and to be worse among patients with both T2D and CA. Though the subjects’ results on Schirmer test, hyperemia quantification, and tear meniscus height did not differ significantly, they were abnormal among all T2D patients included. It is important to consider that a increase in these values may be related to a compensatory reflex phase of DED (18, 22, 49).

The results herein strongly suggest that individuals with both T2D and CA experience severe neuropathy in all parts of the body—not only in the foot, but also in the peripheral nerves, the cardiovascular system and the eyes, among other possible systems.

Study limitations to be acknowledged include the cross-sectional design and small sample size. However, the latter is justified by the rarity of CA, even in tertiary hospitals. The main strengths of this study are the use of a highly specific method for cardiovascular autonomic neuropathy diagnosis (CARTs combined with spectral analysis of the HRV) and the systematic ocular assessment provided by a broad panel of tests.

In summary, our data suggest that, due to their association with established cardiovascular autonomic neuropathy and peripheral neuropathy, dry eye disease symptoms and ocular findings could be considered additional clinical tools in the screening and follow-up treatment of diabetic neuropathy and related complications.

## Data Availability Statement

The raw data supporting the conclusions of this article will be made available by the authors, without undue reservation.

## Ethics Statement

The studies involving human participants were reviewed and approved by Institutional Research Ethics Committee Board (CAAE 56897416.9.0000.5404) of the School of Medical Sciences, University of Campinas (UNICAMP), Campinas, São Paulo, Brazil. The patients/participants provided their written informed consent to participate in this study. The patients/participants provided their written informed consent to participate and publish their figures in this study.

## Author Contributions

All authors listed have made a substantial, direct, and intellectual contribution to the work, and approved it for publication.

## Funding

MA has a research grant from the São Paulo Research Foundation (FAPESP) 2014/19138-5. DEZ-W has a research grant from National Council for Scientific and Technological Development (CNPq) 302827/2018-8. This study was financed in part by the Coordenação de Aperfeiçoamento de Pessoal de Nível Superior - Brasil (CAPES) - Finance Code 001.

## Conflict of Interest

The authors declare that the research was conducted in the absence of any commercial or financial relationships that could be construed as a potential conflict of interest.

## References

[1] Dahlström E Sandholm N. Progress in Defining the Genetic Basis of Diabetic Complications. Curr Diabetes Rep (2017) 17(9):1–13. 10.1007/s11892-017-0906-z 28779365

[2] Cavanagh PR Young MJ Adams JE Vickers KL Boulton AJM. Radiographic abnormalities in the feet of patients with diabetic neuropathy. Diabetes Care (1994) 17(3):201–9. 10.2337/diacare.17.3.201 8174448

[3] Dalla Paola L. Confronting a dramatic situation: The charcot foot complicated by osteomyelitis. Int J Low Extrem Wounds (2014) 13(4):247–62. 10.1177/1534734614545875 25123373

[4] Ramanujam CL Stapleton JJ Zgonis T. Diabetic charcot neuroarthropathy of the foot and ankle with osteomyelitis. Clin Podiatr Med Surg (2014) 31:487–92. 10.1016/j.cpm.2013.12.001 25281510

[5] Dimitropoulos G Tahrani AA Stevens MJ. Cardiovascular autonomic neuropathy in patients with diabetes mellitus. World J Diabetes (2014) 5(1):17–39. 10.4239/wjd.v5.i1.17 24567799 PMC3932425

[6] O’Brien I McFadden JP Corrall R. The influence of autonomic neuropathy on mortality in insulin-dependent diabetes. Q J Med (1991) 79(290):495–502. 10.1093/oxfordjournals.qjmed.a068570 1946930

[7] Rogers LC Frykberg RG Armstrong DG Boulton AJM Edmonds M Ha Van G. The Charcot foot in diabetes. Diabetes Care (2011) 34(9):2123–9. 10.2337/dc11-0844 PMC3161273 21868781

[8] Schmidt BM Holmes CM. Updates on Diabetic Foot and Charcot Osteopathic Arthropathy. Curr Diabetes Rep (2018) 18(74):1–11. 10.1007/s11892-018-1047-8 30112582

[9] Holmes C Schmidt B Munson M Wrobel JS. Charcot stage 0: A review and consideratons for making the correct diagnosis early. Clin Diabetes Endocrinol (2015) 1(18):1–12. 10.1186/s40842-015-0018-0 28702236 PMC5471964

[10] Papanas N Maltezos E. Etiology, pathophysiology and classifications of the diabetic Charcot foot. Diabetes Foot Ankle (2013) 4:1–5. 10.3402/dfa.v4i0.20872 PMC3661901 23705058

[11] Milne TE Rogers JR Kinnear EM Martin HV Lazzarini PA Quinton TR. Developing an evidence-based clinical pathway for the assessment, diagnosis and management of acute Charcot Neuro-Arthropathy: A systematic review. J Foot Ankle Res (2013) 6(30):1–12. 10.1186/1757-1146-6-30 23898912 PMC3737070

[12] Johnson-Lynn SE McCaskie AW Coll AP Robinson AHN. Neuroarthropathy in diabetes: pathogenesis of charcot arthropathy. Bone Jt Res (2018) 7(5):373–8. 10.1302/2046-3758.75.BJR-2017-0334.R1 PMC5987696 29922458

[13] Gouveri E. Charcot osteoarthropathy in diabetes: A brief review with an emphasis on clinical practice. World J Diabetes (2011) 2(5):59–65. 10.4239/wjd.v2.i5.59 21691556 PMC3116009

[14] Wanzou JPV Sekimpi P Komagum JO Nakwagala F Mwaka ES. Charcot arthropathy of the diabetic foot in a sub-Saharan tertiary hospital: A cross-sectional study. J Foot Ankle Res (2019) 12(33):1–9. 10.1186/s13047-019-0343-0 31210786 PMC6567465

[15] Moura-Neto A Fernandes TD Zantut-Wittmann DE Trevisan RO Sakaki MH Santos ALG. Charcot foot: Skin temperature as a good clinical parameter for predicting disease outcome. Diabetes Res Clin Pract (2012) 96:e11–4. 10.1016/j.diabres.2011.12.029 22296852

[16] Achtsidis V Eleftheriadou I Kozanidou E Voumvourakis KI Stamboulis E Theodosiadis PG. Dry eye syndrome in subjects with diabetes and association with neuropathy. Diabetes Care (2014) 37(10):e210–1. 10.2337/dc14-0860 25249675

[17] Manaviat MR Rashidi M Afkhami-Ardekani M Shoja MR. Prevalence of dry eye syndrome and diabetic retinopathy in type 2 diabetic patients. BMC Ophthalmol (2008) 8(10):1–5. 10.1186/1471-2415-8-10 18513455 PMC2435518

[18] Stapleton F Alves M Bunya VY Jalbert I Lekhanont K Malet F. TFOS DEWS II Epidemiology Report. Ocul Surf (2017) 15(3):334–65. 10.1016/j.jtos.2017.05.003 28736337

[19] Goebbels M. Tear secretion and tear film function in diabetics. Br J Ophthalmol (2000) 84:19–21. 10.1136/bjo.84.1.19 10611093 PMC1723218

[20] Lv S Cheng J Sun A Li J Wang W Guan G. Mesenchymal stem cells transplantation ameliorates glomerular injury in streptozotocin-induced diabetic nephropathy in rats via inhibiting oxidative stress. Diabetes Res Clin Pract (2014) 104(1):143–54. 10.1016/j.diabres.2014.01.011 24513119

[21] Dogru M Katakami C Inoue M. Tear function and ocular surface changes in noninsulin-dependent diabetes mellitus. Ophthalmology (2001) 108(3):586–92. 10.1016/S0161-6420(00)00599-6 11237914

[22] Bron AJ de Paiva CS Chauhan SK Bonini S Gabison EE Jain S. TFOS DEWS II pathophysiology report. Ocul Surf (2017) 15(3):438–510. 10.1016/j.jtos.2017.05.011 28736340

[23] Benítez-Del-Castillo JM Acosta MC Wassfi MA Diáz-Valle D Gegúndez JÁ Fernandez C. Relation between corneal innervation with confocal microscopy and corneal sensitivity with noncontact esthesiometry in patients with dry eye. Investig Ophthalmol Vis Sci (2007) 48(1):173–81. 10.1167/iovs.06-0127 17197530

[24] Khan A Petropoulos IN Ponirakis G Menzies RA Chidiac O Paquier J. Corneal confocal microscopy detects severe small fiber neuropathy in diabetic patients with Charcot neuroarthropathy. J Diabetes Investig (2018) 9:1167–72. 10.1111/jdi.12806 PMC6123035 29380548

[25] Herlyn A Prakasam RK Peschel S Allgeier S Kohler B Winter K. Corneal subbasal nerve plexus changes in severe diabetic Charcot foot deformity: a pilot study in search for a DNOAP biomarker. J Diabetes Res (2018) 2018:5910639. 10.1155/2018/5910639 30525053 PMC6247393

[26] Belmonte C Nichols JJ Cox SM Brock JA Begley CG Bereiter DA. TFOS DEWS II pain and sensation report. Ocul Surf (2017) 15(3):404–37. 10.1016/j.jtos.2017.05.002 PMC5706540 28736339

[27] Craig JP Nichols KK Akpek EK Caffery B Dua HS Joo C. TFOS DEWS II Definition and Classification Report. Ocul Surf (2017) 15(3):276–83. 10.1016/j.jtos.2017.05.008 28736335

[28] Baudouin C Messmer EM Aragona P Geerling G Akova YA Benítez-del-Castillo J. Revisiting the vicious circle of dry eye disease: A focus on the pathophysiology of meibomian gland dysfunction. Br J Ophthalmol (2016) 100(3):300–6. 10.1136/bjophthalmol-2015-307415 PMC4789719 26781133

[29] American Diabetes Association 11. Microvascular Complications and Foot Care: Standards of Medical Care in Diabetes-2020. Diabetes Care (2020) 43(Suppl.1):S135–51. 10.2337/dc20-S011 31862754

[30] American Diabetes Association. Screening for Type 2 Diabetes. Clin Diabetes (2000) 18(2):69. 10.2337/diacare.25.2007.S33

[31] Parisi MCR Godoy-Santos AL Trevisan Ortiz R Sposeto RB Sakaki MH Nery M. Radiographic and functional results in the treatment of early stages of Charcot neuroarthropathy with a walker boot and immediate weight bearing. Diabetes Foot Ankle (2013) 4:1–5. 10.3402/dfa.v4i0.22487 PMC3813827 24179634

[32] Wolffsohn JS Arita R Chalmers R Djalilian A Dogru M Dumbleton K. TFOS DEWS II Diagnostic Methodology report. Ocul Surf (2017) 15(3):539–74. 10.1016/j.jtos.2017.05.001 28736342

[33] Arita R Itoh K Maeda S Maeda K Furuta A Fukuoka S. Proposed Diagnostic Criteria for Obstructive Meibomian Gland Dysfunction. Ophthalmology (2009) 116(11):2058–2063.e1. 10.1016/j.ophtha.2009.04.037 19744718

[34] Robertson D Biaggioni I Burnstock G Low PA Paton JFR. Primer on the Autonomic Nervous System. (2012). pp. 693–703. 10.1016/C2010-0-65186-8

[35] Young MJ Boulton AJM Macleod AF Willians DRR Sonksen PH. A multicentre study of the prevalence of diabetic peripheral neuropathy in the United Kingdom hospital clinic population. Diabetologia (1993) 36:150–4. 10.1007/bf00400697 8458529

[36] Vinik AI Erbas T Casellini CM. Diabetic cardiac autonomic neuropathy, inflammation and cardiovascular disease. J Diabetes Investig (2013) 4(1):4–18. 10.1111/jdi.12042 PMC3580884 23550085

[37] Acharya UR Joseph KP Kannathal N Lim CM Suri JS. Heart rate variability: A review. Med Biol Eng Comput (2006) 44(12):1031–51. 10.1007/s11517-006-0119-0 17111118

[38] Pop-Busui R Boulton AJM Feldman EL Bril V Freeman R Malik RA. Diabetic neuropathy: A position statement by the American Diabetes Association. Diabetes Care (2017) 40(1):136–54. 10.2337/dc16-2042 PMC6977405 27999003

[39] Vinik AI Ziegler D. Diabetic cardiovascular autonomic neuropathy. Circulation (2007) 115(3):387–97. 10.1161/CIRCULATIONAHA.106.634949 17242296

[40] Spallone V Ziegler D Freeman R Bernardi L Frontoni S Pop-Busui R. Cardiovascular autonomic neuropathy in diabetes: clinical impact, assessment, diagnosis, and management. Diabetes Metab Res Rev (2011) 27:639–53. 10.1002/dmrr 21695768

[41] Antonetti DA Barber AJ Bronson SK Freeman WM Gardner TW Jefferson LF. Diabetic retinopathy: Seeing beyond glucose-induced microvascular disease. Diabetes (2006) 55(9):2401–11. 10.2337/db05-1635 16936187

[42] Kern TS Engerman RL. Vascular lesions in diabetes are distributed non-uniformly within the retina. Exp Eye Res (1995) 60(5):545–9. 10.1016/S0014-4835(05)80069-7 7615020

[43] Srinivasan S Pritchard N Vagenas D Edwards K Sampson GP Russell AW. Retinal Tissue Thickness is Reduced in Diabetic Peripheral Neuropathy. Curr Eye Res (2016) 41(10):1359–66. 10.3109/02713683.2015.1119855 26928267

[44] Yang Y Yu Y Yao K. Pupillary dysfunction in type 2 diabetes mellitus to refine the early diagnosis of diabetic autonomic neuropathy. Neuro-Ophthalmology (2006) 30(1):17–21. 10.1080/01658100600599527

[45] Tekin K Sekeroglu MA Kiziltoprak H Doguizi S Inanc M Yilmazbas P. Static and dynamic pupillometry data of healthy individuals. Clin Exp Optom (2018) 101(5):659–65. 10.1111/cxo.12659 29356077

[46] Wang Y Zekveld AA Naylor G Ohlenforst B Jansma EP Lorens A. Parasympathetic nervous system dysfunction, as identified by pupil light reflex, and its possible connection to hearing impairment. PLoS One (2016) 11(4):1–26. 10.1371/journal.pone.0153566 PMC4835104 27089436

[47] Alves M de C Carvalheira JB Módulo CM Rocha EM. Tear film and ocular surface changes in diabetes mellitus. Arq Bras Oftalmol (2008) 71(6):96–103. 10.1590/s0004-27492008000700018 19274419

[48] Yoon KC Im SK Seo MS. Changes of tear film and ocular surface in diabetes mellitus. Korean J Ophthalmol (2004) 18(2):168–74. 10.3341/kjo.2004.18.2.168 15635831

[49] Schargus M Geerling G. The “wet” dry eye. Ophthalmologe (2009) 106(3):235–41. 10.1007/s00347-008-1908-7 19242698

